# Serum from patients with cirrhosis undergoing liver transplantation induces permeability in human pulmonary microvascular endothelial cells *ex vivo*

**DOI:** 10.3389/fmed.2024.1412891

**Published:** 2024-07-03

**Authors:** Michael P. Bokoch, Fengyun Xu, Krishna Govindaraju, Elliot Lloyd, Kyle Tsutsui, Rishi P. Kothari, Dieter Adelmann, Jérémie Joffre, Judith Hellman

**Affiliations:** ^1^Department of Anesthesia & Perioperative Care, University of California, San Francisco, San Francisco, CA, United States; ^2^Department of Anesthesiology & Perioperative Medicine, Thomas Jefferson University, Philadelphia, PA, United States; ^3^Centre de Recherche Saint-Antoine INSERM U938, Sorbonne University, Paris, France; ^4^Medical Intensive Care Unit, Saint Antoine University Hospital, APHP, Sorbonne University, Paris, France

**Keywords:** acute-on-chronic liver failure, electric cell-substrate impedance sensing, endothelial barrier, ischemia–reperfusion injury, postoperative multiple organ dysfunction, postreperfusion syndrome, transendothelial resistance

## Abstract

**Introduction:**

Patients with cirrhosis undergoing liver transplantation frequently exhibit systemic inflammation, coagulation derangements, and edema, indicating endothelial dysfunction. This syndrome may worsen after ischemia–reperfusion injury of the liver graft, coincident with organ dysfunction that worsens patient outcomes. Little is known about changes in endothelial permeability during liver transplantation. We hypothesized that sera from these patients would increase permeability in cultured human endothelial cells *ex vivo*.

**Methods:**

Adults with cirrhosis presenting for liver transplantation provided consent for blood collection during surgery. Sera were prepared at five time points spanning the entire operation. The barrier function of human pulmonary microvascular endothelial cells in culture was assessed by transendothelial resistance measured using the ECIS ZΘ system. Confluent cells from two different endothelial cell donors were stimulated with human serum from liver transplant patients. Pooled serum from healthy men and purified inflammatory agonists served as controls. The permeability response to serum was quantified as the area under the normalized resistance curve. Responses were compared between time points and analyzed for associations with clinical characteristics of liver transplant patients and their grafts.

**Results:**

Liver transplant sera from all time points during surgery-induced permeability in both endothelial cell lines. The magnitude of permeability change was heterogeneous between patients, and there were differences in the effects of sera on the two endothelial cell lines. In one of the cell lines, the severity of liver disease was associated with greater permeability at the start of surgery. In the same cell line, serum collected 15 min after liver reperfusion induced significantly more permeability as compared to that collected at the start of surgery. Early postreperfusion sera from patients undergoing living donor transplants induced more permeability than sera from deceased donor transplants. Sera from two exemplary cases of patients on preoperative dialysis, and one patient with an unexpectedly long warm ischemia time of the liver graft, induced exaggerated and prolonged endothelial permeability.

**Discussion:**

Serum from patients with cirrhosis undergoing liver transplantation induces permeability of cultured human pulmonary microvascular endothelial cells. Increased endothelial permeability during liver transplantation may contribute to organ injury and present a target for future therapeutics.

## 1 Introduction

Liver transplantation (LT) remains the only curative treatment for patients with end-stage liver disease. There is a global shortage of suitable organs, rising waitlist mortality, and growing demand for LT (1). While the overall number of liver transplants is increasing, the high number of patients awaiting transplants has driven the transplant community to expand the number of eligible grafts. Efforts to expand the donor pool have led to increased utilization of liver grafts from extended criteria donors (2) and an uptick in living donor transplantation (3). Grafts from extended criteria donors, such as those with advanced age, liver steatosis, or donors after circulatory determination of death, may result in more severe hepatic ischemia–reperfusion (IR) injury that leads to postoperative organ failure and poor patient outcomes (4). Vascular endothelial dysfunction is believed to promote organ failure after LT through poorly understood mechanisms (5). Preventing organ failure downstream of liver IR injury is of paramount importance to the field of transplantation. The contribution of endothelial injury to IR injury and organ failure after LT remains to be elucidated.

The endothelium plays a critical role in the regulation of coagulation, inflammation, and vascular permeability. These processes are frequently dysregulated in end-stage liver disease and implicate abnormal endothelial function. A rebalanced coagulation system, with features of both hypo- and hypercoagulability, is a cardinal feature of cirrhosis (6). Inflammatory markers are upregulated in decompensated cirrhosis (7). The severe syndrome of acute-on-chronic liver failure, which is characterized by multiorgan failure and high mortality, is associated with profound systemic inflammation and immune dysfunction and shares some features with sepsis (8). Little is known about alterations in endothelial barrier function along this spectrum of liver disease, although decompensated cirrhosis is frequently accompanied by tissue edema and ascites. Liver reperfusion during transplantation, defined by blood flow restoration through the portal vein, triggers a profound and complex cascade of events that lead to inflammation (9, 10), complement activation (11), and release of damage-associated molecular patterns (5). The resultant mediators may, through their actions on the endothelium, further dysregulate the vascular barrier leading to interstitial edema and organ failure.

We tested the hypothesis that sera isolated from patients undergoing LT (LT sera) would disrupt the barrier function of human microvascular endothelial cells. We cultured monolayers of human pulmonary microvascular endothelial cells (HPMECs) with sera collected from patients undergoing LT. Sera collected before and after liver graft reperfusion were assayed for their ability to induce endothelial permeability. Changes in transendothelial resistance (TER) were used as a surrogate for the effect of LT sera on endothelial barrier function, as has been previously described in studies focused on septic shock (12), trauma (13), and COVID-19 (14). Associations between clinical variables, time points during LT surgery, and the magnitude of permeability change were obtained to reveal the dynamic effects of LT on endothelial function.

## 2 Materials and methods

### 2.1 Human subjects and serum collection

Patients scheduled for LT at the University of California, San Francisco, between May 2021 and May 2023 were approached for participation in the study. Eligible patients were at least 18 years of age and undergoing LT from a deceased or living donor, or simultaneous liver-kidney transplantation (SLKT). The exclusion criteria were a lack of decision-making capacity (e.g., due to hepatic encephalopathy or preoperative intubation) or a primary language other than English. Subjects provided written informed consent to donate blood specimens for research. The local Institutional Review Board approved the protocol (#20-30179).

Blood was drawn from an indwelling radial arterial catheter placed for routine clinical monitoring during LT. Blood was collected into serum separator tubes at the following time points during LT: *S1*, start of surgery (after induction of general anesthesia but before administration of corticosteroids); *S2*, end of the dissection phase of LT (before the administration of heparin or inferior vena cava clamping); *S3*, 15 min after reperfusion of the liver graft through the portal vein; *S4*, 60 min after portal vein reperfusion; and *S5*, 120 min after portal vein reperfusion or the end of surgery (whichever came first). Blood was incubated at room temperature for 30–60 min to allow clot formation. The serum was isolated by centrifugation at 1,800 x *g* for 10 min. Control sera were prepared from blood collected by peripheral venipuncture from three healthy male volunteers, aged 20–30 years. Serum aliquots were frozen at −20°C and then transferred to −80°C until use in experiments with HPMECs. Concentrations of interleukin-6 (IL-6) and IL-8 in LT sera were measured by enzyme-linked immunosorbent assays (DuoSet, R&D Systems, Minneapolis, MN).

### 2.2 HPMEC culture

Primary human pulmonary artery microvascular endothelial cells from one male (24 years old) and one female (57 years old) cadaver were purchased (PromoCell GmbH, Heidelberg, Germany) and used at passages 3–6. Cells were cultured at 37°C, 5% CO_2_ using endothelial cell growth media containing 5% fetal bovine serum (EGM-2 MV, Lonza, Walkersville, MD).

### 2.3 Electric cell-substrate impedance sensing measurements and stimulation with human serum

The integrity of the endothelial monolayer was monitored using the Electric Cell-substrate Impedance Sensing (ECIS) ZΘ device (Applied Biophysics Inc., Troy, NY). The ECIS technology measures the electrical impedance to alternating current flow at multiple frequencies across a monolayer of cells cultured on miniature gold electrodes (15). The resistive component of the impedance at 4,000 Hz is established as a measure of endothelial barrier function (14, 16). A decrease in TER is interpreted as an increase in endothelial permeability. ECIS measurements were performed with 96-well electrodes (96W20idf plates, Applied Biophysics, Inc.). Prior to use, the gold electrodes were stabilized by adding freshly prepared, sterile-filtered *L*-cysteine (10 mM) to each well and incubating for 10 min at room temperature. The plate was rinsed with sterile water. Each well was seeded with HPMECs in 300 μL of EGM-2 MV media at a final density of 120,000 cells cm^−2^. The cells were allowed to rest in a sterile hood at room temperature for 30 min after seeding to minimize convective effects and optimize monolayer dispersion (17).^1^ The seeded ECIS plate was then transferred to a dedicated sterile incubator housing the ECIS ZΘ device and cultured at 37°C with 5% CO_2_. Impedance and TER were recorded using ECIS Software v.1.2.215.0 PC (Applied Biophysics, Inc.) at a sampling rate of approximately one measurement every 6 min.

Once HPMECs reached confluency, as evidenced by the TER reaching a plateau (typically 24–36 h after seeding; Supplementary Figure S1), half (150 μL) of the media was removed from each well and replaced with 150 μL of EGM-2 MV containing the human sera or controls. The final concentration of serum from LT patients and controls was 5% v/v. The inflammatory agonists lipopolysaccharide (LPS) from *E. coli* O113:H10 (final concentration 200 ng mL^−1^, gift of H. Shaw Warren) and recombinant human tumor necrosis factor (TNF)-α (final concentration 10 ng mL^−1^; R&D Systems, Minneapolis, MN) were used as positive controls to induce HPMEC permeability. A 50% media change (EGM-2 MV with no agonist) served as a negative control. Stimulation with human serum was performed in triplicate wells for each patient and time point (*S1*–*S5*). Positive and negative controls were performed in duplicate. It was necessary to transfer the ECIS plates from the 37°C incubator to a biosafety cabinet at room temperature for 3–5 min to allow for the sterile exchange of media. Resistance measurements were interrupted during this time. After stimulation, cells were cultured for 18 h, and TER was measured repeatedly until the end of the experiment.

### 2.4 ECIS data analysis

In pilot experiments, we observed that LT serum consistently induced an increase in HPMEC permeability (decrease in TER), whereas pooled healthy human serum induced a decrease in permeability (increase in TER) within the first 3 h of treatment (Supplementary Figure S1). Therefore, we defined the metric of interest as the difference in the area under the curve (AUC) between the ECIS recordings of HPMECs stimulated with LT serum and healthy pooled human serum between 0 and 3 h after adding sera to wells. The difference in AUC is defined as follows:


$$\Delta AUC={AUC}_{LT\;\mathrm{serum}}-{AUC}_{\mathrm{Pooled}\;\mathrm{healthy}\;\mathrm{serum}}$$

Before determining AUC, the ECIS recordings were normalized by dividing each data point by the plateau value of TER of each well of the ECIS plate just prior to serum stimulation. The data processing protocol was prospectively defined and made public on the Protocols.io platform^2^ before starting the final analysis of the dataset. An example of the data transformations and calculation of ΔAUC is shown in Supplementary Figure S1. The ΔAUC calculation represents an extension of simple AUC analysis that has been previously used for quantification of endothelial permeability responses with ECIS (16, 18).

### 2.5 Clinical data extraction

Clinical data on participating subjects were extracted from the local Transplant Outcomes in Anesthesia Database, a data warehouse comprising perioperative information on both liver donors and recipients (19). Data sources for this warehouse include the electronic health record, the Scientific Registry of Transplant Recipients, and a local, prospectively collected liver transplant anesthesia clinical database. The clinical dataset includes perioperative variables such as demographics, diagnoses, laboratory values, fluids, transfusions, medications, and hemodynamics; surgical parameters such as warm and cold ischemia times and inferior vena cava technique; and key donor variables such as donor type (living, deceased, or donor after cardiac death), age, and body mass index. Early allograft dysfunction of the liver was determined by the Olthoff criteria (20). The presence and stage of acute kidney injury were determined according to the International Club of Ascites definition (21), which is a modification of the Kidney Disease Improving Global Outcomes criteria for use in patients with cirrhosis.

### 2.6 Statistics

Clinical variables are summarized as count and percentage if categorical and median (Q1, Q3) if numeric. Clinical characteristics between patients undergoing deceased versus living donor LT were compared using the Mann–Whitney *U*-test or Fisher’s exact test as appropriate. Normalized resistance of cultured HPMECs in ECIS experiments is plotted as the mean (±SD) of replicate wells, and ΔAUC values (see *Section 2.4*) are displayed as violin plots. The ΔAUC values obtained with LT sera at each time point were compared to the value for pooled healthy serum (ΔAUC = 0) using one-sample Wilcoxon signed-rank tests. The ΔAUC for time point *S1* was compared to other time points using Wilcoxon matched-pairs signed-rank tests for all patients who had complete data. Serum cytokine concentrations (IL-6 and IL-8) were compared between time points *S1* and *S3* using Wilcoxon matched-pairs signed-rank tests. The relationship between ΔAUC at time point *S1* and preoperative characteristics was analyzed using simple linear regression. The association of donor type (deceased versus living) with ΔAUC at time points *S1* and *S3* was analyzed by Mann–Whitney *U*-tests. All tests were two-tailed with significance set at α = 0.05. Analyses were performed with GraphPad Prism 10 and Stata 15.

## 3 Results

### 3.1 Characteristics of human subjects

Serum samples from 25 subjects were applied to HPMECs *ex vivo* and included in the analysis (Table 1). The subjects were predominantly men (60%) and of the expected age and body mass index for LT. The etiology of cirrhosis was approximately evenly distributed between viral hepatitis, alcoholic, non-alcoholic steatohepatitis, and autoimmune causes; 12 (48%) of the subjects had hepatocellular carcinoma, and the median (Q1 and Q3) model for end-stage liver disease sodium (MELD-Na) score was 17 (12, 25). Most subjects (84%) were admitted from home for LT. None of the subjects were admitted to the intensive care unit preoperatively, and 2 (8%) were treated with intermittent hemodialysis; 13 (52%) subjects received a graft from a living donor. Normothermic machine perfusion (Organ Care System, TransMedics, Andover, MA, United States) was used to preserve six (50%) of the grafts from deceased donors at the discretion of the transplant surgeon. The remaining grafts from deceased donors, and all living donor grafts, were preserved with static cold storage according to local practice. Perioperative laboratory values including the MELD-Na components are shown in Table 1.

**Table 1:** Patient and graft characteristics.

	Deceased donor	Living donor	Total	*p*-value
*n* (%)	12 (48%)	13 (52%)	25 (100%)	
Recipient characteristics				
Sex, female	4 (33%)	6 (46%)	10 (40%)	0.69
Age at transplant (y)	62 (60, 67)	52 (37, 57)	58 (44, 64)	0.012
Body mass index (kg m^−2^)	26 (24, 29)	25 (22, 31)	26 (23, 29)	0.70
Etiology of liver disease:				
Hepatitis C	3 (25%)	0 (0%)	3 (12%)	0.19
Hepatitis B	2 (17%)	2 (15%)	4 (16%)	
Alcoholic	2 (17%)	3 (23%)	5 (20%)	
NASH	2 (17%)	3 (23%)	5 (20%)	
Other	2 (17%)	0 (0%)	2 (8%)	
PSC	1 (8%)	5 (38%)	6 (24%)	
Hepatocellular carcinoma	8 (67%)	4 (31%)	12 (48%)	0.12
Inpatient before transplant	3 (25%)	1 (8%)	4 (16%)	0.32
Dialysis, preop	2 (17%)	0 (0%)	2 (8%)	0.22
MELD-Na score	20 (12, 31)	17 (12, 20)	17 (12, 25)	0.26
Preoperative laboratories				
Creatinine (mg dL^−1^)	0.92 (0.77, 1.60)	0.75 (0.66, 0.88)	0.81 (0.67, 1.17)	0.086
Total bilirubin (mg dL^−1^)	2.5 (1.0, 10.6)	2.5 (1.7, 5.0)	2.5 (1.2, 7.2)	0.64
INR	1.5 (1.2, 1.8)	1.3 (1.2, 1.6)	1.4 (1.2, 1.7)	0.60
Sodium (mEq L^−1^)	137 (134, 140)	138 (137, 139)	138 (136, 139)	0.51
Hematocrit (%)	30.8 (25.5, 36.8)	31.9 (28.8, 34.8)	31.5 (28.8, 35.0)	0.70
Fibrinogen (mg dL^−1^)	222 (167, 295)	297 (219, 359)	259 (189, 329)	0.097
Platelets (x 10^9^ L^−1^)	101 (52, 171)	124 (71, 202)	106 (68, 189)	0.79
Albumin (g dL^−1^)	3.3 (3.0, 3.7)	2.6 (2.4, 3.1)	3.0 (2.4, 3.4)	0.036
Donor and graft characteristics				
Donor after brain death	9 (75%)	0 (0%)	9 (36%)	<0.001
Donor after cardiac death	3 (25%)	0 (0%)	3 (12%)	
Living donor	0 (0%)	13 (100%)	13 (52%)	
Machine perfusion	6 (50%)	0 (0%)	6 (24%)	0.005
Cold ischemia time (h)	10.4 (8.4, 15.0)	3.0 (1.8, 3.3)	4.3 (2.9, 10.1)	<0.001
Warm ischemia time (min)	34 (31, 38)	32 (28, 39)	33 (29, 39)	0.51
Albumin (5%) infused, mL	1,500 (1,000, 2000)	1,500 (1,000, 2000)	1,500 (1,000, 2000)	0.91
Fresh frozen plasma transfused (units)	8 (4, 16)	9 (2, 12)	8 (4, 15)	0.62

Data are presented as *n* (%) or median (Q1, Q3). NASH, nonalcoholic steatohepatitis; PSC, primary sclerosing cholangitis; MELD-Na, Model for End-stage Liver Disease-sodium; INR, international normalized ratio.

### 3.2 Induction of HPMEC permeability by LT patient serum

Endothelial barrier formation was considered complete once HPMECs had reached a stable resistance and capacitance during ECIS measurements, which occurred at an average (±SD) of 33 (±10) h after seeding on ECIS plates. Typical plateau values for resistance and capacitance were 1,010 (±360) Ω at 4,000 Hz and 12 (±7) nF at 64,000 Hz, respectively. Treatment with the toll-like receptor 4 agonist LPS and the proinflammatory cytokine TNF-α (positive controls) led to decreased TER of both HPMEC lines, consistent with increased monolayer permeability (Supplementary Figure S2). The peak effect of sera on TER occurred approximately 6 h after addition to cells, and the cells demonstrated partial recovery by 18 h. In contrast, stimulation with healthy pooled human serum resulted in an early increase in resistance (Supplementary Figure S2). A 50% media change (negative control) had minimal impact on TER aside from a transient downward deflection that may be due to mild temperature or pH effects caused by removing the ECIS plate from the incubator to add sera and stimulants.

**Figure 1:**
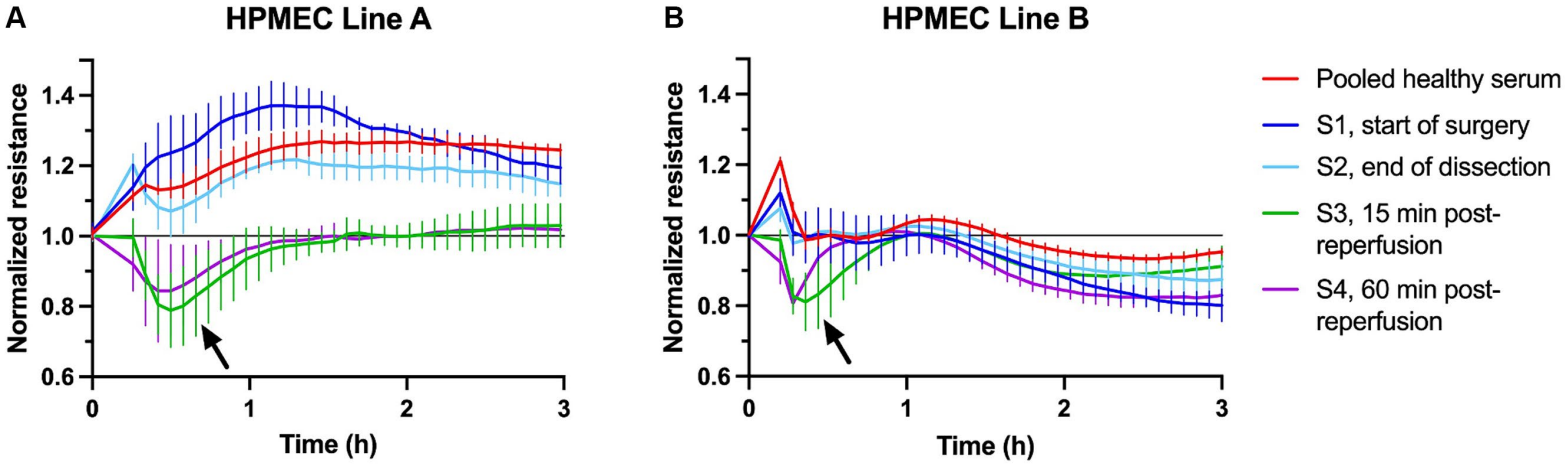
Example plots of transendothelial resistance of human pulmonary microvascular endothelial cells (HPMECs) after stimulation with 5% human serum from healthy subjects (red) or one liver transplant patient (S1–S4, see legend). Measurements were obtained at 4,000 Hz using the ECIS ZΘ system. Resistance was normalized to the plateau value obtained just prior to stimulation at time = 0. **(A)** HPMECs from a 24-year-old male endothelial cell donor. **(B)** HPMECs from a 57-year-old female endothelial cell donor. Each plot is obtained from one experiment with all wells measured simultaneously on a 96-well plate. The error bars indicate the SD of three replicate wells. The arrows indicate the peak permeability effect.

Representative examples of HPMEC permeability triggered by treatment with LT sera are shown in Figure 1. Pooled serum from healthy male control subjects consistently increased TER. A subset of LT sera triggered a decrease in TER consistent with an increase in endothelial permeability. Serum-induced permeability typically recovered within a shorter time as compared to permeability induced by LPS and TNF-α (Supplementary Figure S2). The nadir TER typically occurred approximately 30 min after stimulation with LT sera and persisted for 1–3 h. LT sera-induced changes in TER typically persisted longer in HPMECs from the 24-year-old male endothelial cell donor (line A) as compared to the 57-year-old female donor (line B). Serum collected from some subjects after liver reperfusion (time points *S3* and later) strongly induced HPMEC permeability (Figure 1). In contrast, serum collected from other subjects induced the most permeability at the start of surgery (time point *S1, see below*).

**Figure 2:**
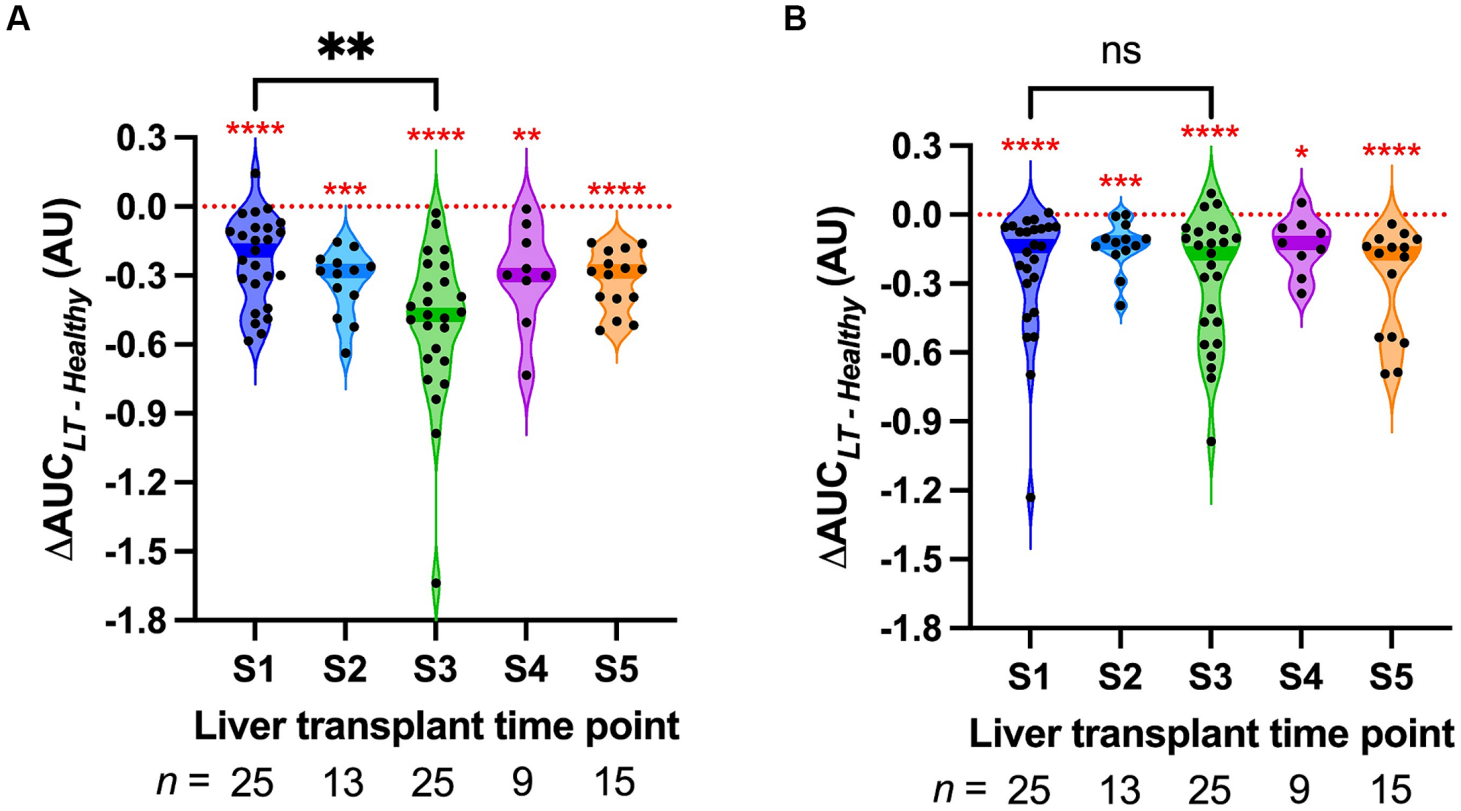
Violin plot of the permeability response (ΔAUC) of human pulmonary microvascular endothelial cells (HPMECs) stimulated with serum from liver transplant patients at the indicated time points. S1, start of surgery; S2, end of the dissection phase; S3, 15 min after portal vein reperfusion; S4, 60 min after portal vein reperfusion; and S5, 120 min after portal vein reperfusion or the end of surgery. Each point represents a different LT serum. The dark-colored bands on the violins indicate the median. The red asterisks indicate the results of the one-sample Wilcoxon signed-rank tests versus zero, the value of ΔAUC that indicates no difference from pooled healthy serum (red dotted line). **(A)** HPMECs from a 24-year-old male endothelial cell donor. **(B)** HPMECs from a 57-year-old female endothelial cell donor. The braces indicate the results of Wilcoxon matched-pairs signed-rank tests between S1 and S3. * *p* < 0.05, ** *p* < 0.01, *** *p* < 0.001, and **** *p* < 0.0001.

The ΔAUC between pooled healthy serum and LT sera was calculated to quantify the ability of LT sera to induce HPMEC permeability (Figure 2). A ΔAUC value of zero indicates that LT sera caused no difference in permeability compared to pooled healthy sera. At every surgical time point for both HPMEC donors, the ΔAUC was significantly different than zero (Figure 2, *red asterisks*). These data indicate that LT sera induce more endothelial permeability than pooled healthy sera (one-sample Wilcoxon signed-rank tests, two-tailed, α = 0.05). Not all LT sera time points were available for each subject due to the limited feasibility of comprehensive collection in the operating room. There was a positive correlation between the ΔAUC of the two HPMEC donor cell lines at the *S1* and *S3* time points (Supplementary Figure S3), indicating that permeability effects induced by a specific LT serum were generally consistent between cell lines.

Sera were obtained from all 25 LT patients at the start of surgery (*S1*) and 15 min after liver reperfusion (*S3*). *S3* sera induced significantly more permeability in HPMEC cell line A as compared to *S1* sera (Wilcoxon matched-pairs signed-rank test, *p* = 0.007, Figure 2A). This difference was not detected in HPMEC cell line B (*p* = 0.75, Figure 2B). Sera from the end of the dissection phase of liver transplant (*S2*), and sera from 2 h after liver reperfusion (*S5*), did not induce significantly more permeability than those from *S1* (Supplementary Figure S4). However, fewer sera samples were available at these time points. In particular, at time point *S3*, serum levels of the inflammatory cytokines IL-6 and IL-8 also increased after liver reperfusion (Supplementary Figure S5).

### 3.3 Association of HPMEC permeability effects with clinical characteristics

**Figure 3:**
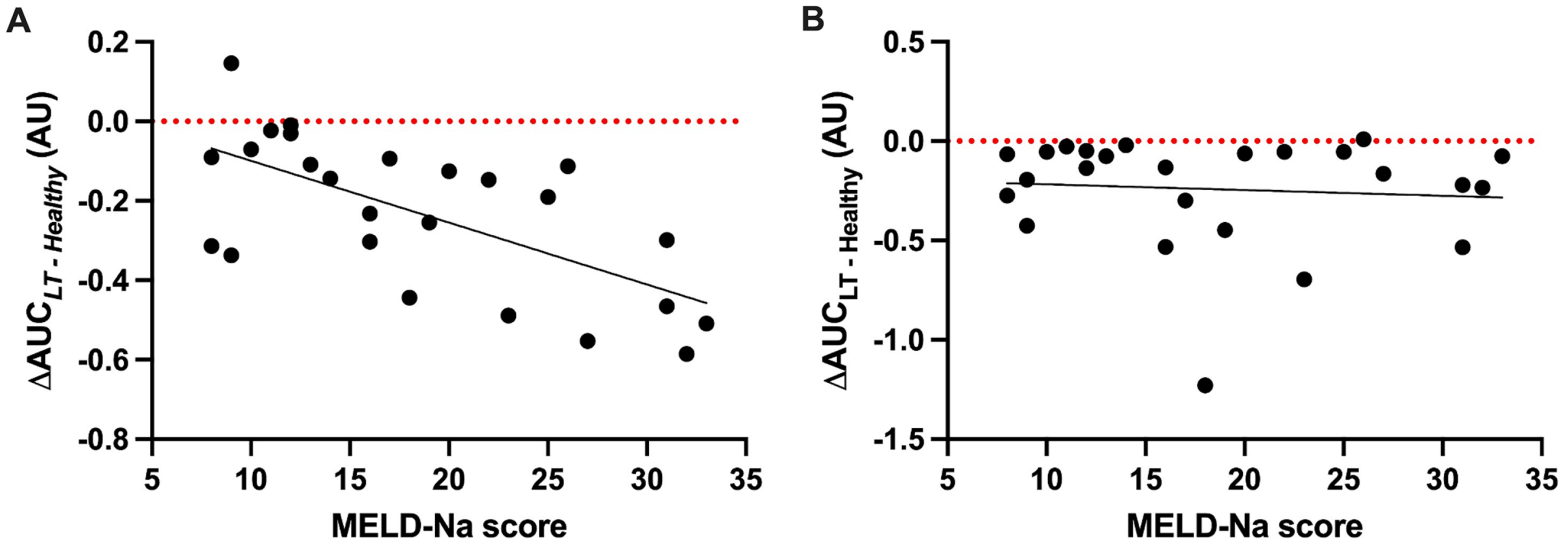
Scatter plots of the permeability response (ΔAUC) of human pulmonary microvascular endothelial cells (HPMECs) to liver transplant sera collected at the start of surgery (S1) as a function of the model for end-stage liver disease—sodium (MELD-Na) score. **(A)** HPMECs from a 24-year-old male endothelial cell donor. There is a negative association between the MELD-Na score and ΔAUC (*F* (1, 23) = 17.29, *p* = 0.0004), with an *R*^2^ of 0.43 and a slope of −0.016 (95% CI -0.023 to −0.008). **(B)** HPMECs from a 57-year-old female endothelial cell donor. There is no association between the MELD-Na score and ΔAUC (*F* (1, 23) = 0.16, *p* = 0.69), with an *R*^2^ of 0.007 and a slope of 0.007 (95% CI -0.018 to 0.012). Black line, best-fit linear regression; red dotted line, value of ΔAUC for serum pooled from healthy adult men.

As LT sera induced significantly more permeability than pooled healthy serum at all time points (Figure 2), we explored the hypothesis that clinical characteristics of patients with cirrhosis would be associated with the HPMEC permeability response to these sera. There was a significant negative association between the MELD-Na score (calculated from the serum sodium, creatinine, total bilirubin, and international normalized ratio) and the ΔAUC for HPMEC cell line A with LT sera from the start of surgery (Figure 3A), indicating that serum from patients with more severe liver disease induced greater permeability in this cell line. This association was not seen in HPMEC cell line B (Figure 3B). In both HPMEC cell lines, there was a significant positive association between the preoperative hematocrit and the ΔAUC (Supplementary Figure S6). There was no evidence for an association between HPMEC permeability response and the sex, age, body mass index, etiology of liver disease, or preoperative serum albumin of the LT patients.

**Figure 4:**
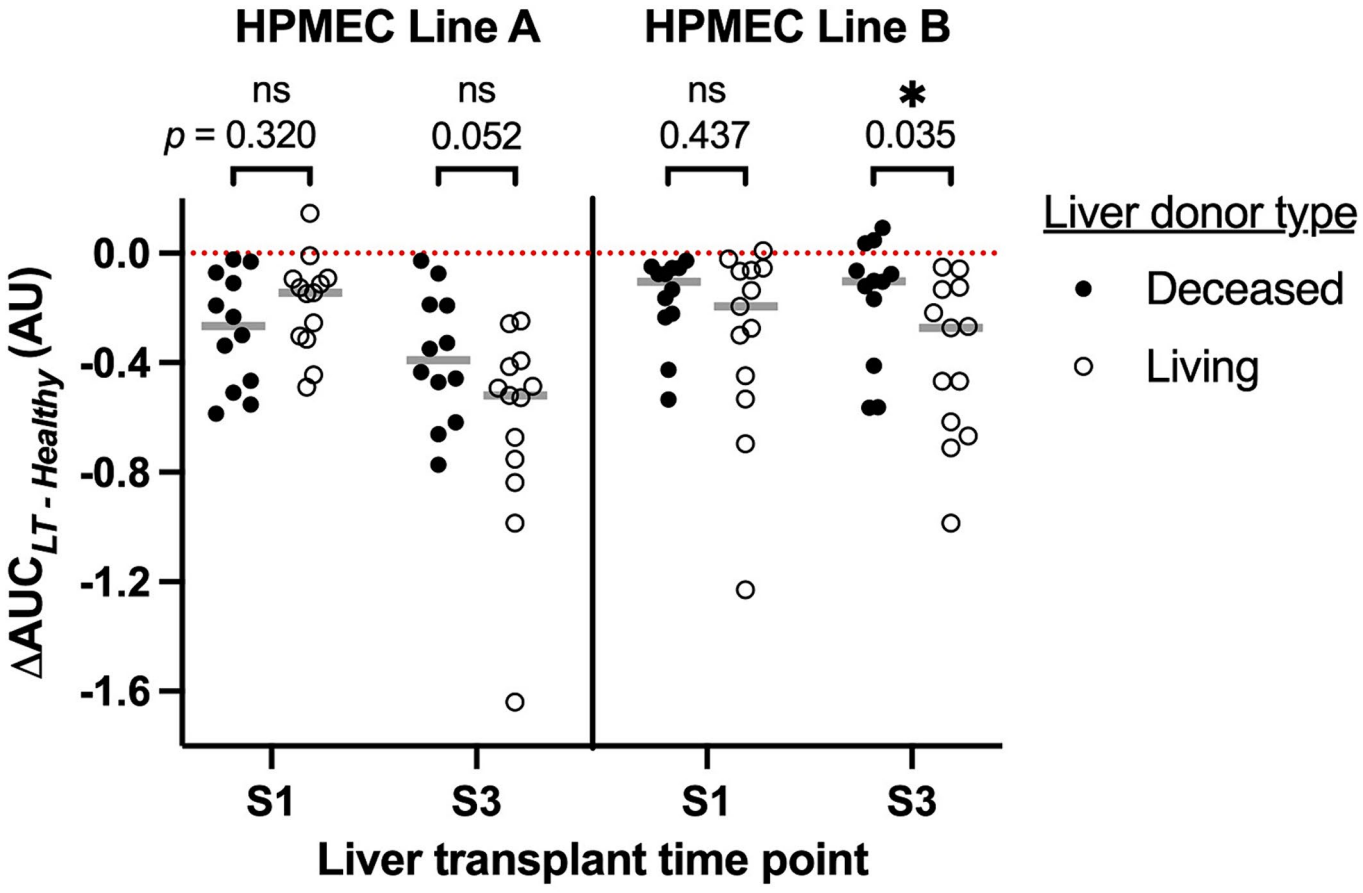
Association of human pulmonary microvascular endothelial cells (HPMEC) permeability response (ΔAUC) with the type of liver donor and time point during liver transplantation. S1, start of surgery; S3, 15 min after portal vein reperfusion. HPMEC line A is derived from a 24-year-old man and HPMEC line B is derived from a 57-year-old woman. The red dotted line indicates the value of ΔAUC for serum pooled from healthy adult men; gray bars indicate the median of each group. Brackets and *p*-values indicate the results of Mann–Whitney *U*-tests.

In total, 13 (52%) of the cohort underwent LT from a living donor. Given the markedly different characteristics of these grafts (typically an isolated right lobe from a healthy donor, with a very short cold ischemia time during which the organ is preserved on ice, Table 1), we investigated the effect of early postreperfusion serum from living donor LT patients on HPMEC permeability (Figure 4). Sera from living donor LT recipients induced significantly more permeability at 15 min after liver reperfusion (*S3*) in HPMEC cell line B as compared to sera from deceased donor recipients (Mann–Whitney *U*-test, *p* = 0.035). In HPMEC cell line A, there was a strong trend toward greater permeability with living donors at *S3* that just missed significance (*p* = 0.052). In particular, living donor LT recipients had milder IR injury measured by the peak serum aspartate aminotransferase level (4) as compared to deceased donor recipients, but no difference was observed in other clinical outcomes (Table 2). No differences in HPMEC permeability based on liver donor type were detected in sera collected from the start of surgery (*S1*).

**Table 2:** Postoperative outcomes.

		Deceased donor	Living donor	*p*-value
	*n* (%)	12 (48%)	13 (52%)	
AST, highest within 72 h after surgery (U L^−1^)		2,658 (1,156, 4,415)	430 (344, 534)	<0.001
Early allograft dysfunction		7 (58%)	4 (31%)	0.24
Postoperative AKI, KDIGO stage	No AKI	2 (17%)	6 (46%)	0.15
	AKI Stage 1	4 (33%)	3 (23%)	
	AKI Stage 2	1 (8%)	3 (23%)	
	AKI Stage 3	5 (42%)	1 (8%)	
ICU length of stay (h)		62 (26–91)	44 (30–47)	0.33
Hospital length of stay (days)		8 (6–12)	8 (6–10)	0.83
Patient status at 1 year after transplant	Alive, graft functioning	12 (100%)	11 (85%)	1.00
	Retransplanted	0 (0%)	1 (8%)	
	Lost to follow-up	0 (0%)	1 (8%)	

Data are presented as *n* (%) or median (Q1, Q3). AST, aspartate aminotransferase; AKI, acute kidney injury; KDIGO, Kidney Disease Improving Global Outcomes; ICU, intensive care unit.

### 3.4 Exemplary cases

In the cohort, there were several extraordinary cases where the clinical characteristics of the patients and their LT procedures were associated with exaggerated HPMEC permeability responses. A brief description of those cases and the endothelial responses at specific time points is presented.

#### 3.4.1 Preoperative dialysis

Two patients in the cohort were on preoperative renal replacement therapy (intermittent hemodialysis) and underwent SLKT. Subject 30 was a 56-year-old man with alcoholic cirrhosis complicated by ascites and acute kidney injury. He was started on hemodialysis 1 day before SLKT. Just before surgery, he had a MELD-Na score of 32, a serum creatinine of 5.28 mg dL^−1^, and a blood urea nitrogen of 136 mg dL^−1^.

Subject 34 was a 70-year-old man with hepatitis C cirrhosis complicated by pulmonary hypertension, ascites, and chronic kidney disease. He was started on hemodialysis 3 weeks before SLKT. Just before surgery, he had a MELD-Na score of 31, a serum creatinine of 4.68 mg dL^−1^, and a blood urea nitrogen of 55 mg dL^−1^.

**Figure 5:**
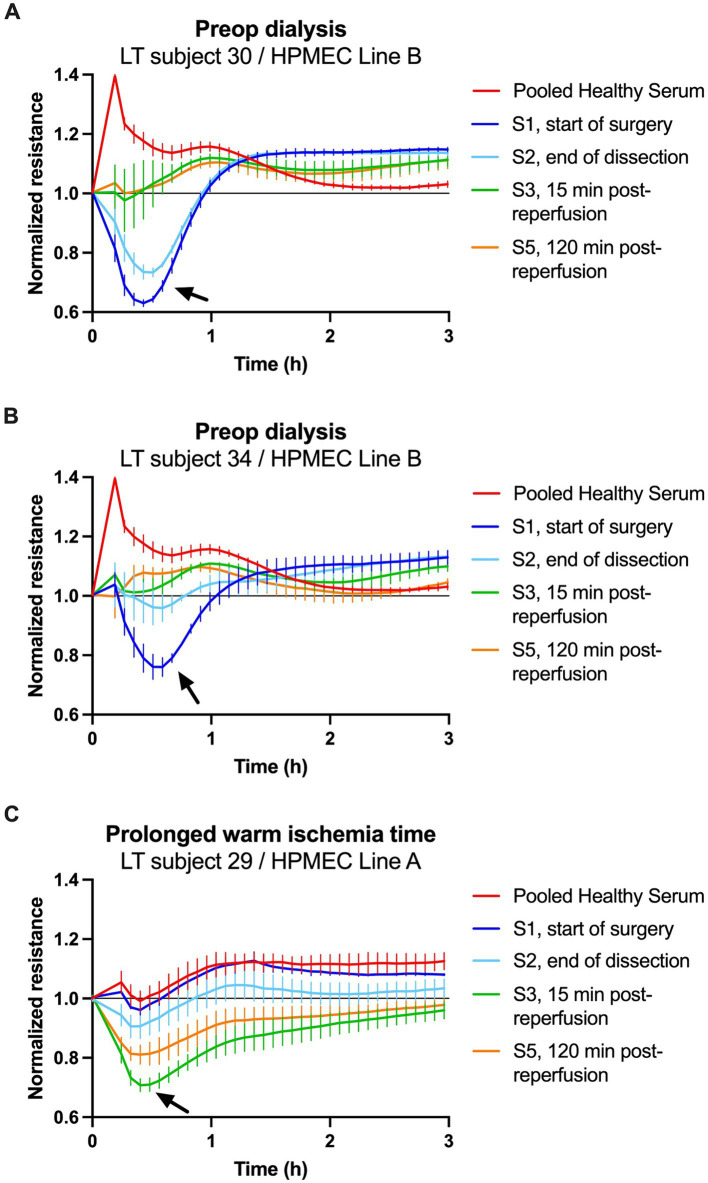
Transendothelial resistance of human pulmonary microvascular endothelial cells (HPMECs) after stimulation with human serum (5%) in patients with notable clinical courses. **(A,B)** Treatment of HPMECs from a 57-year-old female endothelial cell donor with serum from two patients on preoperative dialysis. **(C)** Treatment of HPMECs from a 24-year-old male endothelial cell donor with serum from an LT patient who experienced a 70-min warm ischemia time of the liver graft. The arrows indicate the peak permeability effect.

The start of surgery (*S1*) sera from subjects 30 and 34 strongly induced permeability in HPMEC Line B (Figures 5A,B). These were among the largest ΔAUC observed at *S1*. Postreperfusion sera from subjects 30 and 34 induced minimal permeability. A similar trend was seen with HPMEC Line A but with a smaller magnitude (Supplementary Figures S7A,B). For both subjects, SLKT was straightforward, liver graft function was excellent, intensive care unit stay was short (1–3 days), and dialysis was discontinued postoperatively.

#### 3.4.2 Prolonged warm ischemia time

Subject 29 was a 62-year-old man with class 3 obesity (body mass index 44 kg m^−2^), non-alcoholic steatohepatitis cirrhosis, and hepatocellular carcinoma. He had a MELD-Na score of 10 and underwent LT from a deceased donor. The operation was difficult due to the patient’s body habitus and the large size of the liver graft. As a result, the anhepatic phase was prolonged and the warm ischemia time of the graft was 70 min (99th percentile of all transplants at our center and greater than double the median of the cohort, Table 1). Immediately after liver reperfusion, the patient developed the postreperfusion syndrome manifested by hypotension requiring 250 μg of epinephrine and coagulopathy requiring transfusion of eight units of fresh frozen plasma.

The start of surgery (*S1*) serum from subject 29 did not induce permeability in HPMEC Line A (Figure 5C). However, postreperfusion sera collected at *S3* (15 min) and *S5* (2 h) strongly induced permeability. A similar trend was seen with HPMEC Line B, but the changes in permeability were shorter in duration (Supplementary Figure S7C). This patient’s postoperative course was complicated by early allograft dysfunction of the liver, acute kidney injury requiring renal replacement therapy, and a prolonged stay in the intensive care unit (26 days).

## 4 Discussion

This study demonstrates that serum from patients undergoing LT induces permeability in HPMECs *ex vivo*. LT sera induced more HPMEC permeability than serum from healthy human subjects at all five time points, spanning the entire liver transplant operation. This finding demonstrates that circulating factors present during LT can reduce endothelial barrier function. The severity of liver disease (measured by the MELD-Na score) was positively associated with HPMEC permeability at the start of surgery (*S1* time point), indicating that decompensated cirrhosis yields a circulatory milieu that increases endothelial permeability. Shortly after liver graft reperfusion, LT sera induced more HPMEC permeability than at baseline, suggesting that IR of the liver graft further reduces endothelial barrier function. Postreperfusion effects on the endothelium may contribute to patient outcomes after LT. Disruption of the endothelial barrier may directly lead to tissue edema and organ failure (22, 23). Patient and graft outcomes are significantly worse when these organ injuries complicate LT (24, 25). Endothelial permeability may also coincide with other dysfunctional endothelial responses that modulate inflammation and coagulation, which may also negatively impact the clinical course (26). Elucidating the circulating triggers and downstream pathways by which LT sera induce endothelial permeability could lead to therapeutic strategies to interrupt the cascade and improve outcomes after LT.

Dysfunction of the vascular endothelium is increasingly recognized as a contributor to organ failure in several critical illnesses. For example, septic shock leads to microcirculatory endothelial dysfunction (26) and increased capillary permeability (27). The resultant interstitial edema may contribute to acute kidney injury (23). Acute respiratory distress syndrome, a common complication of sepsis and trauma, is characterized by profound disruption of the endothelial barrier and persistent lung edema (22). Traumatic shock (13) and COVID-19 infection (14) also increase endothelial permeability in *ex vivo* models. The mechanisms may be heterogeneous for each disease. Despite many suggestive clinical findings such as coagulopathy and edema, there exists little direct evidence for pathologic endothelial activation during LT (28). Our findings indicate that most patients with end-stage liver disease presenting for transplant have factors in their blood that can induce endothelial barrier dysfunction at baseline and after graft reperfusion.

Using a model system of *ex vivo* stimulation of HPMECs, we studied the effects of sera collected at precise time points during LT, ranging from the start of surgery until 2 h after liver reperfusion. The use of TER derived from ECIS ZΘ measurements as a surrogate for endothelial barrier function has been described in studies of sepsis, trauma, and COVID-19 (15, 16, 18). Serum from the start of LT (time point *S1*) increased HPMEC permeability (decreased TER) in essentially all patients studied, which contrasts with pooled serum from healthy male donors, which decreased permeability (increased TER). The *S1* time point reflects the patient’s condition before any surgery has taken place. The magnitude of HPMEC permeability induced by *S1* sera was associated with a high MELD-Na score and a low hematocrit at the start of surgery. These results confirm our hypothesis that endothelial barrier function would be disrupted to a greater degree by sera from patients with more severe liver disease.

Two patients with preoperative renal failure showed exaggerated permeability effects at the start of surgery. This suggests that renal insufficiency may impair endothelial barrier function. In particular, serum from the patient with severe azotemia (blood urea nitrogen 136 mg dL^−1^) had the greatest effect. It is unknown whether azotemia is a trigger for endothelial permeability or a marker for another substance that is renally cleared. An alternate plausible explanation is that both acute renal failure and endothelial permeability are manifestations of the intense inflammatory syndrome of ACLF in these patients (8, 29).

We observed dynamic changes in endothelial permeability during LT. Sera collected 15 min after portal vein reperfusion (*S3* time point) induced greater endothelial permeability in one HPMEC cell line (derived from a young male donor) compared to the start of surgery. This was not observed in a second HPMEC cell line derived from an older, female donor. Postreperfusion serum from one patient with an extremely long warm ischemia time (Subject 29) strongly induced permeability in both cell lines. This patient also developed postreperfusion syndrome, early allograft dysfunction of the liver, and acute kidney injury requiring dialysis. These results suggest that hepatic IR injury can reduce endothelial barrier function, and the effect may be prolonged if the injury is severe. We also observed a trend toward greater HPMEC permeability at the *S3* time point in living donor transplant recipients as compared to deceased donors. This finding is surprising and needs to be confirmed in a larger cohort. Ischemia–reperfusion injury tends to be less severe in living donor LT due to smaller grafts (isolated right or left lobe), shorter cold ischemia times, and excellent graft quality.

We found no evidence that the extensive surgery, blood loss, and tissue damage that occur during the dissection phase of LT (*S2* time point) induce additional permeability versus baseline conditions (*S1*). In fact, sometimes endothelial permeability decreased during this time interval (Figure 5B). One explanation for this could be that subjects received moderately large volumes of both albumin and fresh frozen plasma infusions during surgery (Table 1). Both albumin (30) and fresh frozen plasma (31, 32) are proposed to have protective effects on the endothelium and may restore barrier function. The administration of these fluids may have directly counteracted endothelial dysfunction induced during surgery or diluted circulating permeability triggers.

The mechanism(s) by which LT sera induce permeability in HPMECs *ex vivo* remains to be elucidated. It is possible that a circulating protein or set of proteins, such as proinflammatory cytokines (9, 10), damage-associated molecular patterns (5), or nucleic acid species such as microRNAs (33) act as triggers. We observed elevated serum levels of IL-6 and IL-8 at the *S3* time point, consistent with systemic inflammation. However, a limitation of this study is that we did not measure multiplex protein arrays to generate a more comprehensive list of molecules in immune pathways upstream or downstream of endothelial activation. Small molecules may also accumulate at high levels in liver failure. Ammonia and bilirubin have each been suggested to increase the permeability of brain endothelial cells in culture (34, 35). The effect of these molecules on HPMEC permeability is unknown. However, the accumulation of toxic small molecules due to chronic liver disease cannot solely explain our findings, particularly the increase in permeability triggered by early postreperfusion sera. Endothelial cells may also be affected by serum osmolarity (36), which is frequently below normal in end-stage liver disease. We did not measure the osmolarity of our subjects’ serum. This could be a subject of future investigation.

The advantages of this study include a novel *ex vivo* system for studying the permeability effects of sera from human subjects undergoing LT on cultured human endothelial cells. Sera are collected at precise time points relative to liver graft reperfusion. Rich clinical data are available to aid the interpretation of data from cellular assays. We noted differing permeability responses to two different HPMEC cell lines. We have previously shown that HPMECs derived from different donors respond variably to inflammatory agonists (16) and COVID-19 infection (14). While this heterogeneity may have hindered our ability in the current study to detect consistent effects at all time points, it may also help explain the variable postoperative course in LT recipients. Recipient factors and liver graft quality may play a role in post-transplant endothelial dysfunction. In particular, HPMECs isolated from a male endothelial cell donor (cell line A) were more susceptible to induction of permeability 15 min after liver reperfusion compared to HPMECs isolated from a female donor (cell line B). This finding is interesting given the abundant literature on sex-specific responses to acute inflammation, and the trend toward worse outcomes for men afflicted by inflammatory diseases such as COVID-19 (37). This finding needs to be confirmed in future experiments with larger numbers of endothelial cell lines from both male and female donors.

There are some additional limitations to our study. First, the sample size was relatively small. Second, there was incomplete serum collection at some time points. Serum was collected at *S1* and *S3* for all patients, but time points *S2*, *S4*, and *S5* were incompletely sampled. This resulted from logistical barriers to sampling at certain times. For instance, many operations occurred overnight when research personnel were not available to collect and process serum samples. Cases may exceed 10 h in duration. These issues led to our cohort being underpowered to determine associations between endothelial permeability and postoperative outcomes or organ failure. Third, frozen–thawed serum aliquots do not faithfully preserve the biological activity of all molecules circulating in the patient. Labile mediators may be degraded during sample preparation or produced during clot formation in serum separator tubes. A fourth limitation is that we studied HPMECs, but lung endothelium may not be the most relevant model for organ failure after LT. Lung injury is rarely diagnosed after LT (reported incidence <1%). When lung injury occurs after LT, it is usually attributed to transfusion-related acute lung injury and the associated mortality is high (27%) (25). Ischemia–reperfusion of the liver graft may also be a contributor (25, 38). By contrast, acute kidney injury is quite common (up to 80%) (19) after LT and contributes to significant morbidity, mortality, and graft loss (24). Therefore, the kidney endothelium is an interesting potential target for future studies.

This study demonstrates that human serum undergoing LT induces permeability in HPMECs *ex vivo*. The severity of liver disease at the start of surgery was associated with decreased endothelial barrier function. Liver IR yielded a trend toward greater endothelial permeability, but this phenomenon was heterogeneous between HPMEC cell lines. A trend toward greater HPMEC permeability was observed at 15 min after reperfusion of grafts from living donors as compared to deceased donors. Living donor grafts have shorter cold ischemia times and milder IR injury. This finding raises the intriguing possibility that opening of the endothelial barrier after reperfusion may not be entirely harmful, especially if early and transient. For example, an increase in endothelial permeability may facilitate the infiltration of leukocytes into injured tissues, clearance of necrotic cellular debris, and allow inflammation to resolve (39, 40). Elucidating the mechanisms and timing by which LT induces endothelial permeability could identify new therapeutic pathways to target and reduce post-transplant organ failure.

## Data availability statement

The raw data supporting the conclusions of this article will be made available by the authors, without undue reservation.

## Ethics statement

The studies involving humans were approved by University of California, San Francisco Institutional Review Board. The studies were conducted in accordance with the local legislation and institutional requirements. The participants provided their written informed consent to participate in this study.

## Author contributions

MB: Conceptualization, Formal analysis, Funding acquisition, Investigation, Methodology, Project administration, Resources, Validation, Visualization, Writing – original draft, Writing – review & editing. FX: Investigation, Methodology, Writing – review & editing. KG: Investigation, Validation, Visualization, Writing – review & editing. EL: Investigation, Writing – review & editing. KT: Investigation, Writing – review & editing. RK: Data curation, Writing – review & editing. DA: Data curation, Resources, Writing – review & editing. JJ: Conceptualization, Methodology, Writing – review & editing. JH: Conceptualization, Funding acquisition, Project administration, Resources, Supervision, Writing – original draft, Writing – review & editing.

## Footnotes

^1^ https://www.biophysics.com/public/pdf/ECISHandbook.pdf

^2^ https://dx.doi.org/10.17504/protocols.io.bp2l69erklqe/v5
