# Supplementary material for: Serum from patients with cirrhosis undergoing liver transplantation induces permeability in human pulmonary microvascular endothelial cells ex vivo
Source: Front Med (Lausanne). 2024 Jul 3;11:1412891. doi: 10.3389/fmed.2024.1412891 (PMC11252006; doi:10.3389/fmed.2024.1412891)
Supplement: Supplementary file 1 [file Data_Sheet_1.docx]

**Supplementary Material**

**Supplementary Figure 1.** Example of data processing and calculation of ΔAUC.

**Supplementary Figure 2.** Positive and negative ECIS controls.

**Supplementary Figure 3.** Correlation of permeability response between HPMEC cell lines.

**Supplementary Figure 4.** Profile plots of the HPMEC permeability response at different liver transplant time points.

**Supplementary Figure 5.** Serum concentrations of the cytokines IL-6 and IL-8 at different intraoperative time points.

**Supplementary Figure 6.** Association between HPMEC permeability response and preoperative hematocrit for liver transplant sera at the start of surgery.

**Supplementary Figure 7.** Examples of ECIS from patients with notable clinical courses.

**
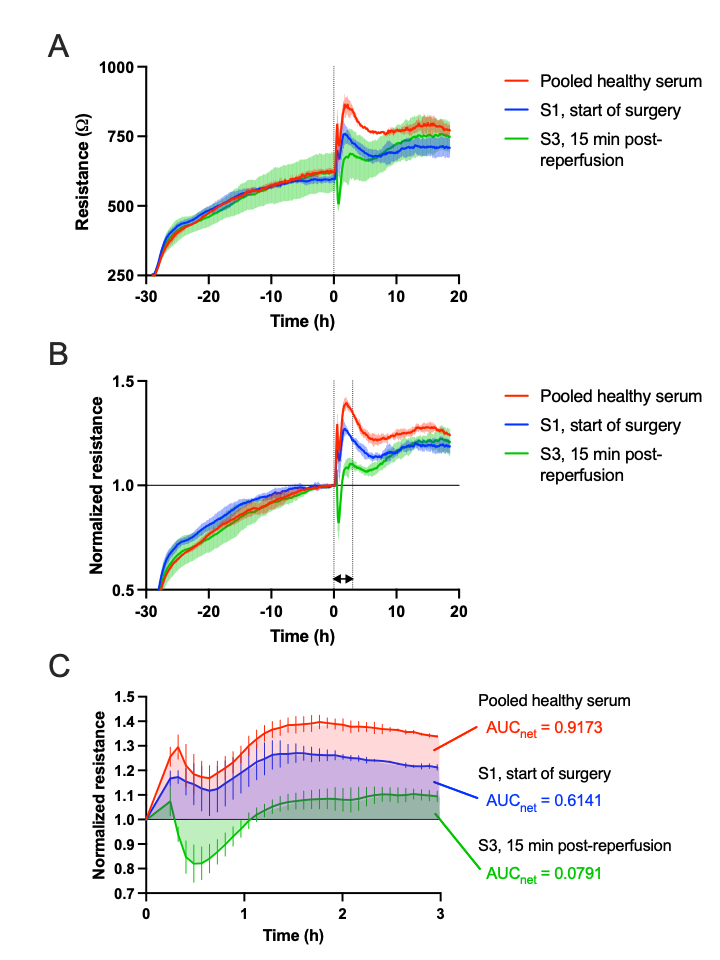
**

**Supplementary Figure 1.** Example of data processing and calculation of ΔAUC for ECIS ZΘ recordings of transendothelial resistance after stimulation with human serum (5%). Error bars indicate the SD of triplicate wells. **(A)** Raw data showing the resistance measured at 4,000 Hz from the time of 96W20idf plate seeding (approximately -29 h) through serum stimulation (0 h, *vertical line*) and the end of the experiment (approximately +18 h). *S1 and S3*, liver transplant sera. **(B)** Normalized resistance for the same period, obtained by dividing all measurements by the resistance immediately prior to serum stimulation. *Double arrows* indicate the region of interest for ΔAUC calculation. **(C)** Area under the curve (AUC) from 0-3 h shown as the shaded region for each curve above and below a normalized resistance of 1.0. Note the *S3* *(green)* curve consists of two positive areas and one negative area, which are summed to give the net AUC. In this example, the ΔAUC are calculated as follows:

ΔAUC = AUC*_LT serum_* – AUC*_Pooled healthy serum_*

ΔAUC*_S1_* = 0.6141 – 0.9173 = -0.3032

ΔAUC*_S3_* = 0.0791 – 0.9173 = -0.8382.

**Supplementary Figure 2.** Permeability response from HPMECs stimulated with positive controls tumor necrosis factor α (TNF-α) and lipopolysaccharide (LPS), as well as a negative control (50% media change). Pooled healthy serum serves as the baseline for comparison of all liver transplant sera. Note that stimulation with healthy serum consistently increases the resistance of the endothelial monolayer above baseline. One representative experiment is shown for each HPMEC cell line. Error bars indicate the SD of triplicate wells. **(A)** HPMECs from a 24-year-old male endothelial cell donor. **(B)** HPMECs from a 57-year-old female endothelial cell donor.

**Supplementary Figure 3.** Correlation of permeability response (ΔAUC) between HPMECs from a 24-year-old male endothelial cell donor (*x-axis*, Line A) and HPMECs from a 53-year-old female endothelial cell donor (*y-axis*, Line B) using sera from liver transplant patients at the time points indicated. *Dashed lines* indicate the best-fit linear regressions. The permeability response of the two cell lines was positively correlated at the start of surgery (*blue* *line*, F(1,23) = 7.721, *p* = 0.01), with an *R*^2^ of 0.25 and a slope of 0.73 (95% CI 0.19 to 1.27), and also 15 min after portal vein reperfusion (*green line*, F(1,23) = 11.64, *p* = 0.002), with an *R*^2^ of 0.34 and a slope of 0.49 (95% CI 0.19 to 0.78).

**Supplementary Figure 4.** Profile plots of the permeability response (ΔAUC) of HPMECs to liver transplant sera at different intraoperative time points. **(A-C)** HPMECs from a 24-year-old male endothelial cell donor. **(D-E)** HPMECs from a 57-year-old female endothelial cell donor. *S1*, start of surgery; *S2*, end of the dissection phase; *S3*, 15 min after portal vein reperfusion; and *S5*, 120 min after portal vein reperfusion or the end of surgery. Wilcoxon matched-pairs signed-rank tests were conducted at each time point for all subjects that had both serum samples present, as indicated by the *n* in each panel. *Red dotted line*, value of ΔAUC for serum pooled from healthy adult males; **, *p*<0.01.

**Supplementary Figure 5.** Serum concentrations of the cytokines **(A)** interleukin-6 (IL-6) and **(B)** interleukin-8 (IL-8) at different intraoperative time points. Box plots are drawn according to the Tukey method. The y-axis of panel B is shown with a break (175 to 500 pg mL^-1^ to allow display of two outliers. *S1*, start of surgery; *S3*, 15 min after portal vein reperfusion; and *S5*, 120 min after portal vein reperfusion or the end of surgery. The number of samples (*n*) measured at each time point is indicated below. Braces indicate the results of Wilcoxon matched-pairs signed rank tests between S1 and S3, because these time points had complete data. * p<0.05, ** p<0.01, *** p<0.001, and **** p<0.0001. While there is a trend towards even higher IL-6 and IL-8 levels at *S5*, statistical testing was not applied due to incomplete data at this time point.

**Supplementary Figure 6.** Association between HPMEC permeability response (ΔAUC) and the preoperative hematocrit for liver transplant sera at the start of surgery. **(A)** HPMECs from a 24-year-old male endothelial cell donor. **(B)** HPMECs from a 57-year-old female endothelial cell donor. In panel *A*, there was a positive association between hematocrit and ΔAUC (*F*(1, 23) = 21.85, *p* = 0.0001), with an *R*^2^ of 0.49 and a slope of 0.024 (95% CI 0.013 to 0.034). In panel *B*, there was also a positive association (*F*(1, 23) = 8.07, *p* = 0.009), with an *R*^2^ of 0.26 and a slope of 0.025 (95% CI 0.007 to 0.043). *Black line*, best-fit linear regression; *red dotted line*, value of ΔAUC for serum pooled from healthy adult males.

**Supplementary Figure 7.** Example plots of transendothelial resistance of human pulmonary microvascular endothelial cells (HPMECs) after stimulation with human serum (5%) in patients with notable clinical courses. **(A, B)** Treatment of HPMECs from a 24-year-old male with serum from two LT patients on preoperative dialysis. **(C)** Treatment of HPMECs from a 57-year-old female with serum from a LT patient who experienced a 70 min warm ischemia time of the liver graft. *Arrows* indicate the peak permeability effect.
